# Targeting centrosome amplification, an Achilles' heel of cancer

**DOI:** 10.1042/BST20190034

**Published:** 2019-09-10

**Authors:** Dorota Sabat-Pośpiech, Kim Fabian-Kolpanowicz, Ian A. Prior, Judy M. Coulson, Andrew B. Fielding

**Affiliations:** 1 Cellular and Molecular Physiology, Institute of Translational Medicine, University of Liverpool, Crown St, Liverpool L69 3BX, U.K.; 2 Division of Biomedical and Life Sciences, Faculty of Health and Medicine, Lancaster University, Lancaster LA1 4YG, U.K.

**Keywords:** cancer, cell invasion, cellular reproduction, centrosomes, therapeutics

## Abstract

Due to cell-cycle dysregulation, many cancer cells contain more than the normal compliment of centrosomes, a state referred to as centrosome amplification (CA). CA can drive oncogenic phenotypes and indeed can cause cancer in flies and mammals. However, cells have to actively manage CA, often by centrosome clustering, in order to divide. Thus, CA is also an Achilles' Heel of cancer cells. In recent years, there have been many important studies identifying proteins required for the management of CA and it has been demonstrated that disruption of some of these proteins can cause cancer-specific inhibition of cell growth. For certain targets therapeutically relevant interventions are being investigated, for example, small molecule inhibitors, although none are yet in clinical trials. As the field is now poised to move towards clinically relevant interventions, it is opportune to summarise the key work in targeting CA thus far, with particular emphasis on recent developments where small molecule or other strategies have been proposed. We also highlight the relatively unexplored paradigm of reversing CA, and thus its oncogenic effects, for therapeutic gain.

## Introduction

Centrosomes are small intracellular organelles, consisting of a pair of centrioles [1] surrounded by ordered layers of pericentriolar material (PCM) [2]. Centrosome number is normally tightly regulated during the cell cycle: cells enter G1 with one centrosome, which is duplicated during S-phase [3]. Centrosomes are the dominant microtubule organising centres in animal cells, nucleating microtubule minus ends [4]. The two centrosomes present during mitosis anchor each half of the bipolar mitotic spindle, thus contributing to the accurate division of chromosomes into each of the newly forming daughter cells [4]. Centrosomes can also organise microtubule minus ends in interphase, aiding cell polarity and contributing to directed cell migration [5].

Centrosome structure and number are often aberrant in cancer [6,7], with CA observed in nearly all tumour types examined [8], as well as in pre-cancerous lesions [9]. Whilst it is beyond the scope of this article to provide a thorough review of the prevalence of CA across tumour types, in order to provide some context to the cancers that may be most amenable to the targeting of CA, the results of two excellent studies on this topic, one on clinical [8] and one on cell line samples [10] are summarised in Table 1. Chan [8] provided a clinical overview of CA in human cancers, where multiple studies across a range of solid and haematological cancers were examined. CA has also been observed in a plethora of cancer cell lines, broadly agreeing with the *in vivo* observations above, although the methods to identify and score CA vary across studies. A step to address this issue was taken in a recent paper where the NCI-60 panel of cell lines was characterised, using a double stain to identify *bona fide* centrioles [10]. It confirmed that CA is common in cancer cell lines from numerous cancer types, including breast, ovarian, central nervous system, colon, skin, non-small cell lung, prostate and renal cancer cells lines as well as cell lines from leukemia, lymphoma and myeloma.

**Table 1:** Summary of two studies on clinical and cell line CA status across cancer types

Cancer type	Clinical data [8]			Cell line data [10]		
	% of samples with CA	Studies	Total *n*	% of cell lines that displayed CA (number from total tested in NCI-60 panel)		Range of % of cells showing amplification within cell lines displaying CA
Solid tumours						
Breast	75–100	18	582	67	(4 out of 6)	14.5–32.6
Neural	89–100	7	181	33	(2 out of 6)	22.2–23.6
Ovarian	78–100	3	91	50	(3 out of 6)	14.8–20.0
Head and neck	62–100	8	193	/	/	/
Urothelial	50–100	9	598	/	/	/
Anogenital	62–100	4	100	/	/	/
Colorectal	65–100	3	132	33	(2 out of 6)	23.9–57.1
Prostate	28–94	4	266	50	(1 out of 2)	16.1
Lung	24–100	3	249	50	(4 out of 8)	25.0–62.1
Bone and soft tissue	18–100	6	165	/	/	/
Adrenal	100	2	14	/	/	/
Hepatobiliary	0–91	2	110	/	/	/
Testicular	33–100	1	36	/	/	/
Pancreatic	0–85	1	16	/	/	/
Renal	25	1	8	29	(2 out of 7)	18.9–30.6
Skin	/	/	/	57	(4 out of 7)	13.7–40.4
Haematological malignancies						
Leukaemia	88–100	7	266	100	(4 out of 4)	19.4–48.2
Lymphomas	41–100	10	195	100	(1 out of 1)	14.3
Myeloma	17–100	3	161	100	(1 out of 1)	26.3

The clinical data summarises some of the key data from Chan [8], a wide-ranging clinical assessment of CA prevalence. The cell line data summarises key findings from Marteil et al. [10] that assessed CA status across the NCI-60 cell panel. In this study, CA was defined as >13% of cells with >4 centrioles, the cut off being determined by analysis of the frequency and variance of CA in tissue-matched non-cancerous cell lines.

These and other studies show that a wide range of cancers exhibit CA. In terms of cancer types where our understanding of the possibilities for therapeutically targeting CA is most advanced, two recent papers suggest CA as a common feature and an attractive therapeutic target for triple-negative breast cancer, which lacks other common targetable features [11,12].

CA is associated with oncogenic phenotypes such as aneuploidy [13,14], increased invasiveness [15,16] and aberrant stem cell divisions [17]. It has also been proposed that CA may allow cancer cells to compensate for cancer mutations that decrease the functionality of centrosomes [18]. Whilst for a long time it was not apparent whether CA was a cause or consequence of cancer it is now clear that, at least under some conditions, CA is sufficient to promote tumorigenesis in flies and mammals [17,19]. Intriguingly, it was recently shown that non-cell-autonomous invasion can be induced by paracrine signalling of cells containing CA [16], suggesting that within heterogeneous tumours, a subpopulation of cells with CA can have far-reaching effects on neighbouring cancer cells. Structural centrosome abnormalities can also induce non-cell-autonomous invasion [20], further highlighting the importance of centrosomes in metastatic processes.

**Figure 1.**
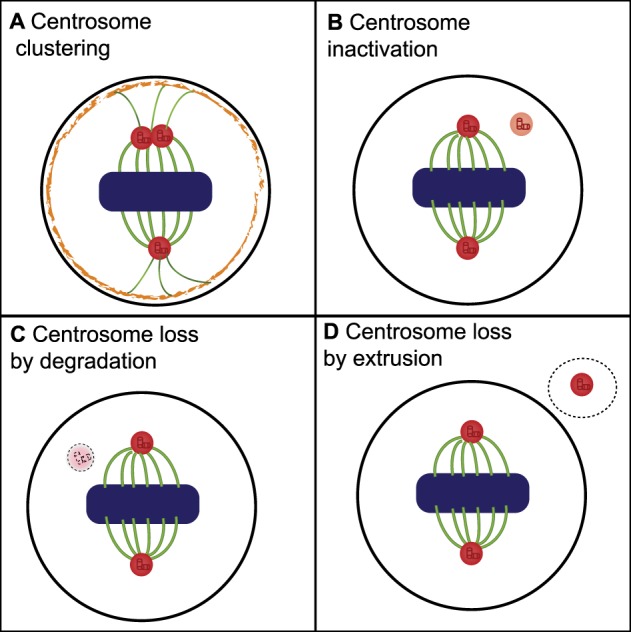
Cellular mechanisms for coping with CA. Cancer cells, with >2 centrosomes in G2 and mitosis, may use one of four mechanisms to prevent dangerous multipolar mitosis. Of these, centrosome clustering is the most well characterised in human cancer cells, with centrosome inactivation also reasonably well-studied in model organisms. Methods to disrupt these mechanisms are discussed and shown in Figure 2.

The potential mechanisms leading to CA in cancer are numerous and include centriole over duplication, *de novo* centrosome formation, fragmentation of overly elongated centrioles and cytokinesis failure [10,21]. When cells enter mitosis with CA, they typically form multipolar mitotic spindles, which if uncorrected invariably lead to cell death. This is either due to prolonged mitotic arrest and apoptosis, or due to division into multiple daughter cells lacking essential genes due to massive aneuploidy that die in the subsequent cell cycle. To avoid these mitotic pitfalls, cells display a number of coping mechanisms (Figure 1). The most commonly observed and well-studied of these is centrosome clustering, whereby the numerous centrosomes are gathered into just two poles during mitosis, allowing pseudo-bipolar mitotic division and cell survival. Other potential coping mechanisms include centrosome inactivation and centrosome loss by either degradation or extrusion (Figure 1). Of these alternative mechanisms, centrosome inactivation is the best-studied in model organisms [17,22,23] although it remains understudied in human cancer cells.

It was proposed several years ago that inhibition of centrosome clustering may exert a cancer-specific therapeutic effect, causing cancer cell death with minimal effect on normal cells displaying two centrosomes [24–26]. Although disruption of centrosome clustering is now widely appreciated as a potential therapeutic strategy in cancer [7,27], as yet no suitably selective interventions have been described. Here, we review the potentially targetable features of CA, including centrosome clustering, but also considering centrosome inactivation and centrosome-dependent invasion. Finally, we examine possible strategies to reverse CA as an alternative therapeutic approach. We aim to highlight approaches where therapeutically relevant small molecule inhibitors are available, with an emphasis on progress made in the last 2 to 5 years. Table 2 lists potential protein targets, categorised according to the mechanisms to which they contribute.

**Table 2:** Proteins that could be disrupted to target cells with CA

Protein target(s)	Target mechanism (subcategory)	Potential therapeutic strategies	Reference(s)
KIFC1/HSET	CC (S/C)	CW069 and AZ82	[12,25,28]
TACC3, ILK and chTOG	CC (S/C)	ILK and TACC3 inhibitors (or via Aurora-A inhibition)	[29]
HURP	CC (S/C)	Via Aurora-A inhibitors	[30,31]
Aurora-A	CC (S/C)	Aurora-A inhibitors	[32]
Hsp72 and Nek6	CC (S/C)	VER-155008 (Hsp70 family inhibitor)	[33]
STAT3, Stathmin, PLK1, y-tubulin	CC (S/C)	Stat3 inhibitor stattic	[34,35]
CP110	CC (S/C)	CDK1 and CDK2 inhibitors	[36,37]
Myo10	CC (S/C and actin)	N/A	[25,38]
Cortical actin/cell adhesion	CC (cortical actin)	Numerous potential	[25]
Cofillin/actin cortex stability	CC (cortical actin)	CP-673451 and crenolanib are Cofillin activators	[39]
Loss of E-cadherin	CC (cortical actin)	Preventing EMT?	[40]
APC/C	CC (SAC)	APC/C inhibitor proTAME	[41]
CPP and Ndc80 complexes	CC (SAC)	Aurora-B inhibitors	[24]
SAC components	CC (SAC)	Numerous potential	[17,25,42]
PARP6	CC	AZ0108	[43,44]
Deubiquitylases	CC (Various)	Various DUB inhibitors	Proposed in [45]
CPAP-tubulin interaction	Inactivation (and CC)	CCB02 disrupts CPAP-tubulin interaction	[46]
Rac1	CDI	Rac1 inhibitors e.g. NSC23766. Arp2/3 inhibitors, e.g. CK-666	[15]
ROS generation, IL-8, Her2 signalling	CDI	Numerous potential	[16]
PLK4	CA	Centrinone/centrinone B	[47,48]
Reactivation of P53 pathway	CA	e.g. Nutlin or PRIMA-1	[49–51]

Arranged by feature of CA that would be targeted (CC, Centrosome clustering; CDI, Centrosome-dependent invasion; CA, Centrosome amplification; S/C, Spindle/Centrosomal proteins; SAC, spindle-assembly checkpoint).

## Proteins required for centrosome clustering

**Figure 2.**
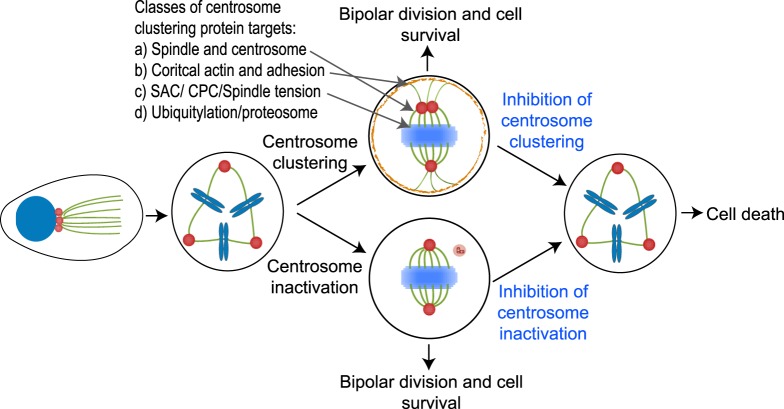
Targeting centrosome clustering or centrosome inactivation as a therapeutic approach. In mitosis, cancer cells cope with CA by clustering or potentially by inactivating extra centrosomes to prevent multipolar mitoses and cell death. Inhibition of these mechanisms (highlighted in blue) will drive cancer cells towards cell death. Four major classes of centrosome clustering proteins, as described in the text, are indicated.

There have been many excellent studies defining proteins required for centrosome clustering, including genome-wide screens in *Drosophila* [25] or human cells [24], and here we consider some of the best-studied and most recent examples. The *Drosophila* screen highlighted three major groups of proteins required for clustering as (i) intrinsic spindle and centrosomal proteins, (ii) proteins required for the organisation of cortical actin and cell adhesion, and (iii) components of the spindle-assembly checkpoint (SAC) [25]. Studies that subsequently identified novel proteins required for clustering in humans, have confirmed that these three mechanisms are widely conserved. Another prominent class of proteins discovered in the *Drosophila* screen were proteins of the ubiquitin/proteasome system, which we also consider below. Figure 2 illustrates the consequences of inhibiting centrosome clustering with reference to these four classes of proteins.

### Intrinsic spindle and centrosomal proteins

The principal hit in the *Drosophila* screen was Ncd, whose human homologue is KIFC1 (kinesin family member C1)/HSET [25]. KIFC1 is a minus-end directed kinesin-14 family member, with a motor domain and an N-terminal microtubule-binding domain enabling it to cross-link neighbouring microtubules and, due to its minus-end activity, focus microtubule minus ends at spindle poles [52]. It also binds to a centrosomal protein CEP215/CDK5RAP2, tethering microtubule minus ends to centrosomes, thus further contributing to spindle pole focusing [28]. It is this pole-focusing activity that is required for centrosome clustering. As well as clustering amplified centrosomes, KIFC1 is required to cluster acentrosomal spindle poles, which are also more common in cancer compared with normal cells [53]. Recently, using an integrated genomic and siRNA functional validation approach, KIFC1 was independently identified as a malignant cell-specific dependency factor in triple-negative breast cancers [12]. These new data have again brought KIFC1 to the fore as a candidate cancer-specific target. Several small-molecule inhibitors against KIFC1 have been developed, including CW069 [54], AZ82 [55,56] and SR31527 [57]. Whilst these first-generation centrosome-declustering agents are valuable lead compounds that specifically induce multipolar spindles in cells with CA and decrease the growth of cancer cells, their overall specificity on cancer cell survival compared with normal cells is somewhat limited [12,54,55,57]. Although the reasons for the limited specificity of these compounds is not fully understood, it seems likely to be a pharmacological issue of these first-generation inhibitors, rather than a biological problem of targeting KIFC1, as several studies have demonstrated a cancer-specific requirement for KIFC1 when using siRNA mediated KIFC1 knockdown [12,25,53]. It is hoped that subsequent generations of KIFC1 inhibitors, perhaps aided by novel KIFC1-screening platforms [58], will provide a broader therapeutic window. Whilst much interest has focused on KIFC1, it is necessary but not sufficient for centrosome-clustering [40], so the discovery of other proteins required in this process is highlighting new therapeutic targets, in some cases where drugs or more specific inhibitors are already available.

ILK (integrin-linked kinase), a centrosomal protein that regulates the complex formation of the K-fibre associated proteins TACC3 and chTOG/CKAP5 [59,60] is also required for centrosome clustering [29]. Whilst all poles of multipolar spindles resulting from ILK or TACC3 depletion contain centrioles, suggesting they result from the de-clustering of amplified centrosomes, chTOG/CKAP5 knockdown causes extensive spindle multipolarity due to the appearance of acentrosomal spindle poles [29–62]. These results suggest ILK or TACC3 are good candidates for specific therapeutic targets, whilst CKAP5/ch-TOG would not be specific to cells displaying CA. Of note, recently developed TACC3 inhibitors [63,64], have not yet been tested in the context of CA.

NEK6 phosphorylation of HSP72 is required to cluster amplified centrosomes, whilst their depletion in non-cancer derived cells does not affect spindle formation or mitotic progression, thus making them attractive cancer-specific targets [33]. Hsp72 is required for localisation of the TACC3/ch-TOG complex to K-fibres [65], again demonstrating the importance of this unit, and hence stable K-fibres, for centrosome clustering. Of additional interest from a therapeutic standpoint, the potent HSP70 family inhibitor VER-155008, produced cancer-specific spindle multipolarity [33], with specific Nek6 inhibitors also under development.

HURP is another microtubule-associated protein that stabilises K-fibres in mitosis and is required for clustering of amplified centrosomes in cancer cells [30]. HURP is also required for meiotic spindle assembly in mouse oocytes, where bipolar spindles must form in the absence of canonical centrosomes. Interestingly, KIFC1 [66–68] and TACC3 [69,70] are also required for acentrosomal spindle assembly in multiple organisms, suggesting that cancer cells may be dependent on these meiotic mechanisms to form bipolar spindles when the centrosome complement of the cell is abnormal. Aurora-A is also required for clustering [32]. This is particularly noteworthy as Aurora-A is a mitotic kinase required for both TACC3 spindle localisation [71,72] and HURP activity [31]. As several Aurora-A inhibitors are already in advanced clinical trials [73], this could be a particularly fruitful route to therapeutic inhibition of centrosome clustering.

CP110 is a centrosomal protein whose mitotic phosphorylation by CDK2 is required to prevent multipolar spindle formation [36]. Hence CDK2 inhibition also provides a strategy to prevent centrosome clustering, although, with broad-ranging roles, this may not specifically target cells with CA [37].

Whilst screens for compounds that induce centrosome-declustering have the drawback that the mechanism is initially unknown, a major advantage is that the therapeutic potential is intrinsically validated by the identification of the small molecule inducing the phenotype of interest. This is the case for a series of screens for chemical inhibitors of centrosome clustering that identified static, a STAT3 inhibitor [34,35]. Whilst STAT3 is widely known to regulate transcription [74], its effect on centrosome clustering is transcription-independent. In this scenario, STAT3 works via a Stathmin-PLK1 cascade that up-regulates gamma-tubulin levels at centrosomes, allowing centrosomes to nucleate a dense network of microtubules during mitosis and hence become clustered [34].

### Cortical actin and cell adhesion proteins

The second major class of proteins required for centrosome clustering were those involved in the organisation of cortical actin and cell adhesion, including proteins that link the actin and microtubule cytoskeletons [25] and these findings have also been born out in more recent studies. A prominent example of this class of centrosome-clustering protein is the unconventional myosin MYO10. MYO10 can directly bind astral microtubules and co-ordinate their positioning with subcortical actin, thus mediating the alignment of mitotic spindles in relation to actin-rich retraction fibres [38]. This centrosomal-positioning function of MYO10 means it is also required for the clustering of amplified centrosomes [25,38].

Cofilin is an actin severing protein [75]. A recent screen identified two small molecules (CP-673451 and crenolanib) that lead to disruption of cortical actin, the formation of multipolar spindles and cell death in cancer cells, by activating cofilin [39]. These experiments not only reinforce the importance of actin organisation in centrosome clustering, but the small molecules displayed CA-specific effects on multipolar spindle induction at therapeutically-relevant doses, highlighting their promise as potential treatments in cancer. Cortical contractility was recently highlighted as a requirement for centrosome clustering [40]. Epithelial cells that express E-Cadherin have low cortical contractility and are inherently poor at clustering centrosomes. Loss of E-Cadherin, whether experimentally induced or occurring in cancer cells that have undergone epithelial to mesenchymal transition, increases cortical contractility, limiting the movements of supernumerary centrosomes, which in turn allows them to be clustered by a KIFC1-dependent mechanism [40].

### Spindle-assembly checkpoint/chromosomal passenger complex/spindle tension proteins

SAC function was identified as a major requirement for centrosome clustering in the *Drosophila* screen [25], allowing cells to remain in prometaphase/metaphase until centrosomes are effectively clustered and a pseudo-bipolar spindle has formed. The human genome-wide screen also identified numerous SAC components, including the chromosomal passenger complex (CPC) [24]. Hits from the latter study reinforced the importance of co-ordination of the actin and microtubule skeletons, and the general requirement of spindle tension for successful centrosome positioning and clustering. If spindle tension is disrupted by any mechanism, including a reduction in chromatid cohesion, mis-attachment of kinetcochores to K-fibres, reduced microtubule generation/stability, or disturbed microtubule-centrosome attachment, then centrosome clustering is inhibited [24]. Although knockdown of some proteins required for spindle tension also produced acentriolar multipolar spindles in non-cancer cells (e.g. FAM29A, HEI-C and HAUS3, members of the augmin complex), depletion of many components, including all members of the CPC (Aurora-B, INCENP, Survivin and Borealin) lead to centriole-containing, cancer-specific multipolar mitoses. This highlights the potential for many drivers of spindle tension to be cancer-specific therapeutic targets.

### Ubiquitylation and proteasomal system proteins

Ubiquitylation is a reversible post-translational modification involving the addition of the 8 kDa protein ubiquitin to target proteins. This can affect protein stability, localisation, or activity. Ubiquitin can be added as a single moiety or different types of ubiquitin chains, which play key roles in many cellular processes including cell signalling and the cell cycle, and often target proteins for degradation by the 26S proteasome. The Anaphase-promoting complex/cyclosome (APC/C) is one of two key cell-cycle ubiquitin ligases and is critical for degradation of multiple proteins at mitotic exit once the SAC is satisfied. Depletion or inhibition of the APC/C by the small molecule proTAME induces centrosome declustering [41]. The mechanism relies on APC/C-CDH1-dependent degradation of the motor kinesin EG5 post-metaphase. When the APC/C is inhibited, EG5 levels are abnormally high in the next metaphase, which leads to an imbalance of spindle forces, disrupting the movement of spindle poles relative to each other during prometaphase and metaphase. Without this movement, cells are rendered unable to cluster amplified centrosomes. APC/C inhibition by proTAME resulted in multipolar spindles solely in cells displaying CA, suggesting this could also be a cancer-specific therapeutic approach [41]. In addition to ubiquitin ligases such as the APC/C, it is also worth considering their counterparts, the deubiquitylase (DUB) family.

DUBs are a family of enzymes that catalyse the removal of ubiquitin from protein substrates. As a druggable protein family whose expression is often dysregulated in cancer, they present attractive new therapeutic targets. We recently reviewed the roles for DUBs in regulating the centrosome cycle, including evidence for their roles in clustering [45]. Briefly, in the *Drosophila* genome-wide screen, two DUBs whose human homologues are USP8 and USP31 were identified [25]. Ubiquitin related proteins were also prominent hits in the human genome-wide screen and included the predicted non-catalytically active DUB USP54 [24]. However, in both cases these classes of proteins were not followed up further, representing an opportunity for discovering a new class of centrosome-clustering regulators. We are currently using targeted DUB siRNA screens to specifically pursue this question. In addition to the hits from the earlier genome-wide screens, some mechanisms via which DUBs may act on centrosome clustering are already apparent. For example, USP33 deubiquitylates the centrosomal protein CP110, regulating centrosome biogenesis [76] and USP33 depletion has a co-operative effect with CDK2 on the CP110-dependent clustering function mentioned earlier [77]. There are also a number of DUBs involved in the regulation of SAC and APC/C activity, including USP4, USP9X, USP39 and USP44 [78–81] which, as discussed above, are required for centrosome clustering.

### Further centrosome clustering targets

Although most centrosome clustering targets can be classed in the above categories, there are some cases where the mechanism of action on clustering is currently not clear. For example, inhibition of Protein mono-ADP-ribosyltransferase 6 (PARP6) with the small molecule AZ0108 induces multipolar spindle formation and apoptosis in a subset of cells *in vitro* [43,44]. The favourable pharmacokinetic properties of this molecule, along with its anti-tumour effects in *in vivo* models, make it another promising agent to induce centrosome declustering in cancer.

Whilst centrosome clustering has been studied in the most detail to date, it is becoming apparent that cancer cells employ other strategies to deal with CA.

## Centrosome inactivation

An alternative method to cope with CA is to ‘inactivate’ extra centrosomes, by reducing their microtubule nucleating capacity during mitosis, so that they do not contribute to spindle formation and therefore do not cause chromosome missegregation (Figure 2). In *Drosophila*, neuroblasts cope with experimentally induced CA by a combination of clustering and inactivation. Centrosome clustering occurs during prophase and prometaphase, with any centrosomes that are not clustered subsequently becoming inactivated [17]. However, in *Drosophila* wing-disc epithelial cells overexpressing *Sak* (the Drosophila orthologue of human PLK4) and therefore displaying CA, the Ezrin–Radixin–Moesin (ERM) protein Moesin is expressed, which localises to centrosomes and maintains the microtubule-nucleating capacity of non-clustered centrosomes, leading to multipolar spindles [22]. As neither clustering nor inactivation mechanisms are completely efficient in these epithelial cells with CA, they become aneuploid and subsequently generate tumours. From a therapeutic perspective, it is not clear whether Moesin activation or inhibition would be beneficial as Moesin expression increased potentially catastrophic multipolar formation, yet was linked to tumourgenesis. Given that the ERM protein family is more complex in humans [82], and that Moesin is often overexpressed in cancers [83], a complete understanding of human models would be required to delineate the best therapeutic approach. An additional complication to understanding its role in centrosome organisation is that Moesin also plays a role in determining cortical contractility [84,85], which as described above, influences centrosome clustering [40].

Recently, the importance of the interaction between Centrosomal-P4.1-associated protein (CPAP) and beta-tubulin in centrosome function has been examined [46]. CPAP normally limits PCM recruitment to centrosomes; hence genetic disruption of the interaction between CPAP and tubulin lead to high PCM recruitment and enhanced microtubule nucleation prior to mitosis. This, in turn, resulted in the disruption of centrosome clustering. A screen for small molecules that disrupt CPAP binding to tubulin identified CCB02, that leads to centrosome de-clustering, prolonged multipolar mitosis and cell death of cancer cells with CA, both *in vitro* and *in vivo* [46].

## Reversing oncogenic effects of CA as a therapeutic intervention

**Figure 3.**
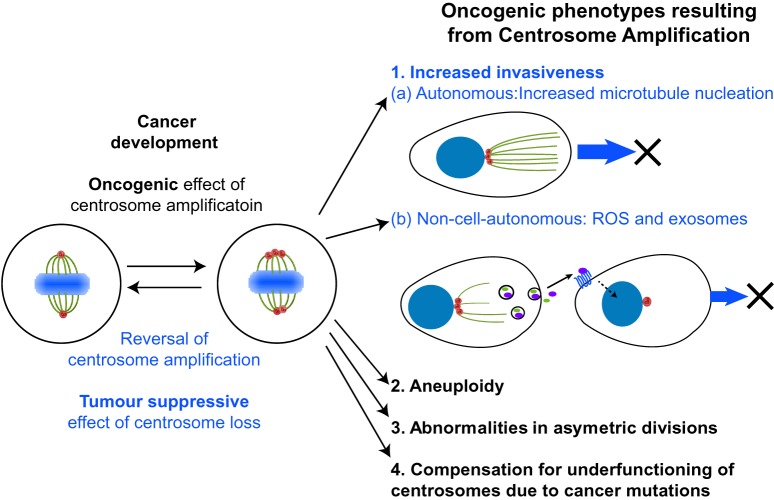
Reversing the oncogenic effects of CA as a therapeutic approach. CA causes several oncogenic phenotypes. Potential methods to target some of these phenotypes or to reverse CA (highlighted in blue) are discussed in the text.

In the sections above, we have discussed the coping mechanisms on which cells with CA rely in order to be able to divide, and considered how these may be disrupted for therapeutic gain. However, CA also induces oncogenic phenotypes, including increased cell invasiveness [15,16], low-level aneuploidy [13], errors in asymmetric cell division[17] and, potentially, allowing cells to compensate for under-functioning centrosomes due to cancerous mutations [18] (Figure 3). We will now discuss how these oncogenic phenotypes could be inhibited, firstly by examining how one of the phenotypes, centrosome-dependent invasion, may be manipulated and secondly examining the potential of reversing CA in cancer cells to negate oncogenic phenotypes.

### Centrosome-dependent invasion

A major finding in the field was that CA potentiates cell invasion via a microtubule nucleating, RAC1-dependent, effect [15]. Both RAC1 itself and the downstream ARP2/3 complex can be inhibited with small molecules, presenting a clear therapeutic route to decrease CA-dependent invasion [15]. Furthermore, it has recently been discovered that CA can also induce a non-cell autonomous increase in invasive capacity. The mechanism involves a CA-dependent increase in reactive oxygen species inducing secretion of pro-invasive factors such as IL-8, which in turn activate pro-invasive pathways, such as HER2 signalling, in neighbouring cells [16]. Potentially, multiple aspects of this pathway could be inhibited to lead to a therapeutic advantage [16].

### Reversal of centrosome amplification

Given that CA induces aggressive oncogenic phenotypes [13,15–17], there is a compelling argument that reversal of CA could also have therapeutic effects.

To reverse amplification, we first need to identify what causes CA in cancer. There are numerous potential routes to CA, including cytokinesis failure, centriole over duplication, *de novo* centrosome formation and fragmentation of overly elongated centrioles [10,21]. Whilst it is not well defined which of these processes lead to CA in particular cancer types, there is good evidence that these mechanisms do occur in cancers. For example, cytokinesis failure leading to tetraploid intermediates is well documented in tumours [86,87]. At the molecular level, two major pathways, loss of the tumour suppressor P53 and overexpression of PLK4, are the most studied to date. It was shown over two decades ago that CA develops in *P53* knockout cells [88], and P53 loss of function has since been observed in many cancers displaying CA [89–92]. In P53-null cells, dysregulated CDK2 and CDK4 activity lead to CA via premature progression through the centrosome replication cycle [93]. Generation of cells with CA may not, however, be as straightforward as simply losing normal P53 function. In a study of CA on the developing brain, *P53*^−/−^ mice had normal centrosome number [94]. Additionally, a study of the NCI-60 cell line panel revealed no significant difference in CA between cells with functional or mutated P53, and only half of the cell lines with mutated P53 displayed CA [10]. On balance, it seems that P53 loss is permissive of, but not sufficient to induce, CA. In order to attempt to reverse CA in patients, there are several therapeutic routes to reactivate the P53 pathway; Nutlins and PRIMA-1 are small molecule inhibitors that target P53 through two different mechanisms. Nutlin and its analogues, some of which are in phase I and II trials, target the interaction between P53 and one of its regulatory proteins, MDM2 [49]. Overexpression of MDM2, most notably in haematological cancers, leads to low levels of P53 [95]. If cells with CA are indeed dependent on ablation of Wildtype-P53 activity to maintain their proliferative ability, treatment with Nutlin could be a viable option to reverse CA. Another approach that could be used to reverse CA via P53 is PRIMA-1 and its analogue, PRIMA-1^MET^. These molecules, currently in phase II and III trials, target Y220C mutant P53 to restore wild type function [50,51].

Overexpression of PLK4, a key protein involved in centrosome replication, has been used to experimentally induce CA [96,97]. PLK4 is overexpressed in numerous cancer types [98–102], although its expression does not always correlate with CA [48]. In relation to the P53 findings described above, in a study of PLK4-induced CA in mice, CA lead to reduced proliferative ability that could be rescued by P53 knock-down. Tumours that developed as a result of CA generally had reduced P53 target gene expression [19]. Taken together, the evidence suggests PLK4 overexpression is sufficient but not necessary to induce CA. In addition, normal PLK4 function is required for centriole duplication and therefore to maintain CA in any system, not just in those with PLK4 overexpression. Therefore, it may be possible to inhibit PLK4 in cancers with or without PLK4 overexpression to reverse CA, and tools to inhibit PLK4 have already been developed. For example, Centrinone was developed as a selective and reversible small-molecule inhibitor of PLK4 and was found to deplete centrosomes in cell lines with varying levels of CA [47]. Centrinone has since been used as a tool to investigate centrosome biology and been tested for its therapeutic potential in treating cancer [103–105].

Although P53 reactivation and PLK4 inhibition may be applicable methods to reverse CA in some cases, the key challenge in reversing CA will be to identify the most effective molecular pathways to target. Ongoing advances in omics' technologies should benefit future studies into targetable mechanisms behind CA.

## Perspectives

### Importance of the field

CA is oncogenic via a number of mechanisms but is also a potential therapeutic target. Given that CA is prevalent in a wide range of cancers, including hard to treat cancers such as triple-negative breast cancer [11], exploiting it to kill or decrease the aggressiveness of cancer cells could be very valuable. Recent studies demonstrating that CA can have non-cell autonomous oncogenic effects [16] further broadens the potential therapeutic utility of targeting cells with CA.

### Summary of current thinking

We highlight four ways in which CA could be targeted; by inhibition of centrosome clustering, inhibition of centrosome inactivation, inhibition of centrosome-dependent invasion and finally by reversing CA itself, thereby reversing the oncogenic phenotypes associated with CA. As Table 2 shows, there are good potential routes to inhibit many of these processes therapeutically, as small molecule inhibitors to disrupt many of these mechanisms are available. Despite these exciting developments, no approaches specifically designed to inhibit centrosome clustering have yet reached clinical trials. However, several small molecules that target CA, directly or indirectly, for example, Aurora-A, Aurora-B and CDK inhibitors are in clinical trials as mitotic inhibitors [73] and so in the future, it will be exciting to see if these are particularly beneficial to patients with tumours displaying CA. In terms of selecting these patients, recent advances have been made towards clinically relevant detection of CA in tumours, including the development of the ‘pericentrin abnormality score', an IHC method to detect CA in breast cancers [12], which now requires validation on an independent cohort and other cancer types.

### Future directions

In terms of mitotic coping mechanisms for CA, centrosome clustering is well-studied and several potential targets required for clustering described in this article. Centrosome inactivation is another coping mechanism, that is reasonably well-studied in model organisms but understudied in human cancer. Given this is another potential route to disrupt cells with CA, further study of centrosome inactivation in cancer is certainly merited.

One note of caution on inhibiting centrosome clustering or centrosome inactivation as a therapeutic approach is that, if clustering or inactivation are dampened but not completely inhibited, this could potentially result in a higher frequency of low-level aneuploidy emerging [22], which could drive rather than inhibit tumour aggressiveness. Therefore, careful dosing and/or combination treatments to ensure robust inhibition will be required.

As discussed, several mechanisms are required for centrosome clustering, which raises the possibility that inhibiting more than one mechanism in a combination therapy approach may induce the required robust de-clustering. Although numerous possibilities remain to be explored in this context, the finding that clustering is a two-step process relying firstly on cortical contractility, followed by KIFC1 function [40], suggest that combining cortical actin modulators with inhibition of intrinsic spindle or centrosomal proteins may be a logical starting point for investigating combination therapy approaches.

Whilst this review has focussed on targeting cells with CA, it is also worth noting that multipolar spindle formation can occur in the absence of CA, particularly in cancer cells [53,106] and that the prevention of these multipolar spindles also relies on many of the proteins required for centrosome clustering [53,106,107]. Therefore, there may be added benefit in targeting centrosome clustering mechanisms beyond disrupting cells displaying CA.

In terms of which cancer types may benefit most from the described mechanisms to target CA, much work remains to be done. A good starting point will be to analyse the expression of the protein targets described in this article, to determine cancer types in which they are consistently expressed or overexpressed, and in turn to assess whether those cancer cell types are particularly vulnerable to targeting of the identified proteins. An important example where progress has already been made in this respect is the expression of, and reliance upon, KIFC1 in triple-negative breast cancers [12].

## Abbreviations

APC/C: anaphase-promoting complex/cyclosome
CA: Centrosome amplification
CPAP: centrosomal-P4.1-associated protein
CPC: Chromosomal passenger complex
DUB: Deubiquitylase
K-fibre: Kinetochore fibre
PCM: Pericentriolar material
SAC: Spindle-assembly checkpoint

## References

[1] Loncarek J. and Bettencourt-Dias M. (2018) Building the right centriole for each cell type. J. Cell Biol. 217, 823–835 10.1083/jcb.201704093 29284667 PMC5839779

[2] Fry A.M., Sampson J., Shak C. and Shackleton S. (2017) Recent advances in pericentriolar material organization: ordered layers and scaffolding gels. F1000Res. 6, 1622 10.12688/f1000research.11652.1 29026530 PMC5583744

[3] Nigg E.A. and Holland A.J. (2018) Once and only once: mechanisms of centriole duplication and their deregulation in disease. Nat. Rev. Mol. Cell Biol. 19, 297–312 10.1038/nrm.2017.127 29363672 PMC5969912

[4] Meraldi P. (2016) Centrosomes in spindle organization and chromosome segregation: a mechanistic view. Chromosome Res. 24, 19–34 10.1007/s10577-015-9508-2 26643311

[5] Nigg E.A. and Raff J.W. (2009) Centrioles, centrosomes, and cilia in health and disease. Cell 139, 663–678 10.1016/j.cell.2009.10.036 19914163

[6] Pihan G.A., Purohit A., Wallace J., Knecht H., Woda B., Quesenberry P. et al. (1998) Centrosome defects and genetic instability in malignant tumors. Cancer Res. 58, 3974–3985 PMID:9731511

[7] Godinho S.A. and Pellman D. (2014) Causes and consequences of centrosome abnormalities in cancer. Philos. Trans. R. Soc. Lond. B Biol. Sci. 369, 20130467 10.1098/rstb.2013.0467 25047621 PMC4113111

[8] Chan J.Y. (2011) A clinical overview of centrosome amplification in human cancers. Int. J. Biol. Sci. 7, 1122–1144 10.7150/ijbs.7.1122 22043171 PMC3204404

[9] Pihan G., Zhou Y.N., Purohit A. and Doxsey S.J. (2001) Centrosome defects and genetic instability occur together in precancerous lesions of the breast, cervix and prostate. Mol. Biol. Cell 12, 175A-A

[10] Marteil G., Guerrero A., Vieira A.F., de Almeida B.P., Machado P., Mendonça S. et al. (2018) Over-elongation of centrioles in cancer promotes centriole amplification and chromosome missegregation. Nat. Commun. 9, 1258 10.1038/s41467-018-03641-x 29593297 PMC5871873

[11] Pannu V., Mittal K., Cantuaria G., Reid M.D., Li X., Donthamsetty S. et al. (2015) Rampant centrosome amplification underlies more aggressive disease course of triple negative breast cancers. Oncotarget 6, 10487–10497 10.18632/oncotarget.3402 25868856 PMC4496369

[12] Patel N., Weekes D., Drosopoulos K., Gazinska P., Noel E., Rashid M. et al. (2018) Integrated genomics and functional validation identifies malignant cell specific dependencies in triple negative breast cancer. Nat. Commun. 9, 1044 10.1038/s41467-018-03283-z 29535384 PMC5849766

[13] Ganem N.J., Godinho S.A. and Pellman D. (2009) A mechanism linking extra centrosomes to chromosomal instability. Nature 460, 278–282 10.1038/nature08136 19506557 PMC2743290

[14] Lingle W.L., Barrett S.L., Negron V.C., D'Assoro A.B., Boeneman K., Liu W. et al. (2002) Centrosome amplification drives chromosomal instability in breast tumor development. Proc. Natl Acad. Sci. U.S.A. 99, 1978–1983 10.1073/pnas.032479999 11830638 PMC122305

[15] Godinho S.A., Picone R., Burute M., Dagher R., Su Y., Leung C.T. et al. (2014) Oncogene-like induction of cellular invasion from centrosome amplification. Nature 510, 167–171 10.1038/nature13277 24739973 PMC4061398

[16] Arnandis T., Monteiro P., Adams S.D., Bridgeman V.L., Rajeeve V., Gadaleta E. et al. (2018) Oxidative stress in cells with extra centrosomes drives non-cell-autonomous invasion. Dev. Cell 47, 409–424.e9 10.1016/j.devcel.2018.10.026 30458137 PMC6251975

[17] Basto R., Brunk K., Vinadogrova T., Peel N., Franz A., Khodjakov A. et al. (2008) Centrosome amplification can initiate tumorigenesis in flies. Cell 133, 1032–1042 10.1016/j.cell.2008.05.039 18555779 PMC2653712

[18] Harrison L.E., Bleiler M. and Giardina C. (2018) A look into centrosome abnormalities in colon cancer cells, how they arise and how they might be targeted therapeutically. Biochem. Pharmacol. 147, 1–8 10.1016/j.bcp.2017.11.003 29128368 PMC5733729

[19] Levine M.S., Bakker B., Boeckx B., Moyett J., Lu J., Vitre B. et al. (2017) Centrosome amplification is sufficient to promote spontaneous tumorigenesis in mammals. Dev. Cell 40, 313–322.e5 10.1016/j.devcel.2016.12.022 28132847 PMC5296221

[20] Ganier O., Schnerch D., Oertle P., Lim R.Y., Plodinec M. and Nigg E.A. (2018) Structural centrosome aberrations promote non-cell-autonomous invasiveness. EMBO J. 37, e98576 10.15252/embj.201798576 29567643 PMC5920242

[21] Fukasawa K. (2007) Oncogenes and tumour suppressors take on centrosomes. Nat. Rev. Cancer 7, 911–924 10.1038/nrc2249 18004399

[22] Sabino D., Gogendeau D., Gambarotto D., Nano M., Pennetier C., Dingli F. et al. (2015) Moesin is a major regulator of centrosome behavior in epithelial cells with extra centrosomes. Curr. Biol. 25, 879–889 10.1016/j.cub.2015.01.066 25772448 PMC4386030

[23] Magescas J., Zonka J.C. and Feldman J.L. (2019) A two-step mechanism for the inactivation of microtubule organizing center function at the centrosome. eLife 8, e47867 10.7554/eLife.47867 31246171 PMC6684319

[24] Leber B., Maier B., Fuchs F., Chi J., Riffel P., Anderhub S. et al. (2010) Proteins required for centrosome clustering in cancer cells. Sci. Transl. Med. 2, 33ra38 10.1126/scitranslmed.3000915 20505215

[25] Kwon M., Godinho S.A., Chandhok N.S., Ganem N.J., Azioune A., Thery M. et al. (2008) Mechanisms to suppress multipolar divisions in cancer cells with extra centrosomes. Genes Dev. 22, 2189–2203 10.1101/gad.1700908 18662975 PMC2518815

[26] Rebacz B., Larsen T.O., Clausen M.H., Ronnest M.H., Loffler H., Ho A.D. et al. (2007) Identification of griseofulvin as an inhibitor of centrosomal clustering in a phenotype-based screen. Cancer Res. 67, 6342–6350 10.1158/0008-5472.CAN-07-0663 17616693

[27] Ogden A., Cheng A., Rida P.C., Pannu V., Osan R., Clewley R. et al. (2014) Quantitative multi-parametric evaluation of centrosome declustering drugs: centrosome amplification, mitotic phenotype, cell cycle and death. Cell Death Dis. 5, e1204 10.1038/cddis.2014.164 24787016 PMC4047924

[28] Chavali P.L., Chandrasekaran G., Barr A.R., Tátrai P., Taylor C., Papachristou E.K. et al. (2016) A CEP215-HSET complex links centrosomes with spindle poles and drives centrosome clustering in cancer. Nat. Commun. 7, 11005 10.1038/ncomms11005 26987684 PMC4802056

[29] Fielding A.B., Lim S., Montgomery K., Dobreva I. and Dedhar S. (2011) A critical role of integrin-linked kinase, ch-TOG and TACC3 in centrosome clustering in cancer cells. Oncogene 30, 521–534 10.1038/onc.2010.431 20838383

[30] Breuer M., Kolano A., Kwon M., Li C.-C., Tsai T.-F., Pellman D. et al. (2010) HURP permits MTOC sorting for robust meiotic spindle bipolarity, similar to extra centrosome clustering in cancer cells. J. Cell Biol. 191, 1251–1260 10.1083/jcb.201005065 21173113 PMC3010075

[31] Wu J.-M., Chen C.-T., Coumar M.S., Lin W.-H., Chen Z.-J., Hsu J.T.-A. et al. (2013) Aurora kinase inhibitors reveal mechanisms of HURP in nucleation of centrosomal and kinetochore microtubules. Proc. Natl Acad. Sci. U.S.A. 110, E1779–E1787 10.1073/pnas.1220523110 23610398 PMC3651446

[32] Navarro-Serer B., Childers E.P., Hermance N.M., Mercadante D. and Manning A.L. (2019) Aurora A inhibition limits centrosome clustering and promotes mitotic catastrophe in cells with supernumerary centrosomes. Oncotarget 10, 1649–1659 10.18632/oncotarget.26714 30899434 PMC6422193

[33] Sampson J., O'Regan L., Dyer M.J.S., Bayliss R. and Fry A.M. (2017) Hsp72 and Nek6 cooperate to cluster amplified centrosomes in cancer cells. Cancer Res. 77, 4785–4796 10.1158/0008-5472.CAN-16-3233 28720575

[34] Morris E.J., Kawamura E., Gillespie J.A., Balgi A., Kannan N., Muller W.J. et al. (2017) Stat3 regulates centrosome clustering in cancer cells via Stathmin/PLK1. Nat. Commun. 8, 15289 10.1038/ncomms15289 28474672 PMC5424153

[35] Kawamura E., Fielding A.B., Kannan N., Balgi A., Eaves C.J., Roberge M. et al. (2013) Identification of novel small molecule inhibitors of centrosome clustering in cancer cells. Oncotarget 4, 1763–1776 10.18632/oncotarget.1198 24091544 PMC3858562

[36] Hu S., Danilov A.V., Godek K., Orr B., Tafe L.J., Rodriguez-Canales J. et al. (2015) CDK2 inhibition causes anaphase catastrophe in lung cancer through the centrosomal protein CP110. Cancer Res. 75, 2029–2038 10.1158/0008-5472.CAN-14-1494 25808870 PMC4433598

[37] Kawakami M., Mustachio L.M., Liu X. and Dmitrovsky E. (2018) Engaging anaphase catastrophe mechanisms to eradicate aneuploid cancers. Mol. Cancer Ther. 17, 724–731 10.1158/1535-7163.MCT-17-1108 29559545 PMC6053917

[38] Kwon M.J., Bagonis M., Danuser G. and Pellman D. (2015) Direct microtubule-binding by myosin-10 orients centrosomes toward retraction fibers and subcortical actin clouds. Dev. Cell 34, 323–337 10.1016/j.devcel.2015.06.013 26235048 PMC4672950

[39] Konotop G., Bausch E., Nagai T., Turchinovich A., Becker N., Benner A. et al. (2016) Pharmacological inhibition of centrosome clustering by slingshot-mediated cofilin activation and actin cortex destabilization. Cancer Res. 76, 6690–6700 10.1158/0008-5472.CAN-16-1144 27634760

[40] Rhys A.D., Monteiro P., Smith C., Vaghela M., Arnandis T., Kato T. et al. (2018) Loss of E-cadherin provides tolerance to centrosome amplification in epithelial cancer cells. J. Cell Biol. 217, 195–209 10.1083/jcb.201704102 29133484 PMC5748979

[41] Drosopoulos K., Tang C., Chao W.C. and Linardopoulos S. (2014) APC/c is an essential regulator of centrosome clustering. Nat. Commun. 5, 3686 10.1038/ncomms4686 24751481

[42] Yang Z., Lončarek J., Khodjakov A. and Rieder C.L. (2008) Extra centrosomes and/or chromosomes prolong mitosis in human cells. Nat. Cell Biol. 10, 748–751 10.1038/ncb1738 18469805 PMC2430725

[43] Johannes J.W., Almeida L., Daly K., Ferguson A.D., Grosskurth S.E., Guan H. et al. (2015) Discovery of AZ0108, an orally bioavailable phthalazinone PARP inhibitor that blocks centrosome clustering. Bioorg. Med. Chem. Lett. 25, 5743–5747 10.1016/j.bmcl.2015.10.079 26546219

[44] Wang Z., Grosskurth S.E., Cheung T., Petteruti P., Zhang J., Wang X. et al. (2018) Pharmacological inhibition of PARP6 triggers multipolar spindle formation and elicits therapeutic effects in breast cancer. Cancer Res. 78, 6691–6702 10.1158/0008-5472.CAN-18-1362 30297535

[45] Darling S., Fielding A.B., Sabat-Pośpiech D., Prior I.A. and Coulson J.M. (2017) Regulation of the cell cycle and centrosome biology by deubiquitylases. Biochem. Soc. Trans. 45, 1125–1136 10.1042/BST20170087 28900014 PMC5652225

[46] Mariappan A., Soni K., Schorpp K., Zhao F., Minakar A., Zheng X. et al. (2019) Inhibition of CPAP-tubulin interaction prevents proliferation of centrosome-amplified cancer cells. EMBO J. 38, e99876 10.15252/embj.201899876 30530478 PMC6331730

[47] Wong Y.L., Anzola J.V., Davis R.L., Yoon M., Motamedi A., Kroll A. et al. (2015) Cell biology. Reversible centriole depletion with an inhibitor of Polo-like kinase 4. Science 348, 1155–1160 10.1126/science.aaa5111 25931445 PMC4764081

[48] Denu R.A., Shabbir M., Nihal M., Singh C.K., Longley B.J., Burkard M.E. et al. (2018) Centriole overduplication is the predominant mechanism leading to centrosome amplification in melanoma. Mol. Cancer Res. 16, 517–527 10.1158/1541-7786.MCR-17-0197 29330283 PMC5835182

[49] Burgess A., Chia K.M., Haupt S., Thomas D., Haupt Y. and Lim E. (2016) Clinical overview of MDM2/X-targeted therapies. Front. Oncol. 6, 7 10.3389/fonc.2016.00007 26858935 PMC4728205

[50] Bykov V.J.N., Issaeva N., Shilov A., Hultcrantz M., Pugacheva E., Chumakov P. et al. (2002) Restoration of the tumor suppressor function to mutant p53 by a low-molecular-weight compound. Nat. Med. 8, 282–288 10.1038/nm0302-282 11875500

[51] Sallman D.A., Borate U., Cull E.H., Donnellan W.B., Komrokji R.S., Steidl U.G. et al. (2018) Phase 1/1b study of the stapled peptide ALRN-6924, a dual inhibitor of MDMX and MDM2, as monotherapy or in combination with cytarabine for the treatment of relapsed/refractory AML and advanced MDS with TP53 wild-type. Blood 132, 4066 10.1182/blood-2018-99-118780

[52] She Z.-Y. and Yang W.-X. (2017) Molecular mechanisms of kinesin-14 motors in spindle assembly and chromosome segregation. J. Cell Sci. 130, 2097–2110 10.1242/jcs.200261 28668932

[53] Kleylein-Sohn J., Pöllinger B., Ohmer M., Hofmann F., Nigg E.A., Hemmings B.A. et al. (2012) Acentrosomal spindle organization renders cancer cells dependent on the kinesin HSET. J Cell Sci. 125(Pt 22), 5391–5402 10.1242/jcs.107474 22946058

[54] Watts C.A., Richards F.M., Bender A., Bond P.J., Korb O., Kern O. et al. (2013) Design, synthesis, and biological evaluation of an allosteric inhibitor of HSET that targets cancer cells with supernumerary centrosomes. Chem. Biol. 20, 1399–1410 10.1016/j.chembiol.2013.09.012 24210220 PMC3898838

[55] Wu J., Mikule K., Wang W., Su N., Petteruti P., Gharahdaghi F. et al. (2013) Discovery and mechanistic study of a small molecule inhibitor for motor protein KIFC1. ACS Chem. Biol. 8, 2201–2208 10.1021/cb400186w 23895133

[56] Yang B., Lamb M.L., Zhang T., Hennessy E.J., Grewal G., Sha L. et al. (2014) Discovery of potent KIFC1 inhibitors using a method of integrated high-throughput synthesis and screening. J. Med. Chem. 57, 9958–9970 10.1021/jm501179r 25458601

[57] Zhang W., Zhai L., Wang Y., Boohaker R.J., Lu W., Gupta V.V. et al. (2016) Discovery of a novel inhibitor of kinesin-like protein KIFC1. Biochem. J. 473, 1027–1035 10.1042/BJ20150992 26846349 PMC5488687

[58] Yukawa M., Yamauchi T., Kurisawa N., Ahmed S., Kimura K.-I. and Toda T. (2018) Fission yeast cells overproducing HSET/KIFC1 provides a useful tool for identification and evaluation of human kinesin-14 inhibitors. Fungal Genet. Biol. 116, 33–41 10.1016/j.fgb.2018.04.006 29684553

[59] Fielding A.B., Dobreva I., McDonald P.C., Foster L.J. and Dedhar S. (2008) Integrin-linked kinase localizes to the centrosome and regulates mitotic spindle organization. J. Cell Biol. 180, 681–689 10.1083/jcb.200710074 18283114 PMC2265580

[60] Dobreva I., Fielding A., Foster L.J. and Dedhar S. (2008) Mapping the integrin-linked kinase interactome using SILAC. J. Proteome Res. 7, 1740–1749 10.1021/pr700852r 18327965

[61] Gergely F., Draviam V.M. and Raff J.W. (2003) The ch-TOG/XMAP215 protein is essential for spindle pole organization in human somatic cells. Genes Dev. 17, 336–341 10.1101/gad.245603 12569123 PMC195983

[62] Barr A.R. and Gergely F. (2008) MCAK-independent functions of ch-Tog/XMAP215 in microtubule plus-end dynamics. Mol. Cell Biol. 28, 7199–7211 10.1128/MCB.01040-08 18809577 PMC2593372

[63] Campo L. and Breuer E.-K. (2018) Inhibition of TACC3 by a small molecule inhibitor in breast cancer. Biochem. Biophys. Res. Commun. 498, 1085–1092 10.1016/j.bbrc.2018.03.125 29555478

[64] Yao R., Kondoh Y., Natsume Y., Yamanaka H., Inoue M., Toki H. et al. (2014) A small compound targeting TACC3 revealed its different spatiotemporal contributions for spindle assembly in cancer cells. Oncogene 33, 4242–4252 10.1038/onc.2013.382 24077290

[65] O'Regan L., Sampson J., Richards M.W., Knebel A., Roth D., Hood F.E. et al. (2015) Hsp72 is targeted to the mitotic spindle by Nek6 to promote K-fiber assembly and mitotic progression. J. Cell Biol. 209, 349–358 10.1083/jcb.201409151 25940345 PMC4427782

[66] Mountain V., Simerly C., Howard L., Ando A., Schatten G. and Compton D.A. (1999) The kinesin-related protein, HSET, opposes the activity of Eg5 and cross-links microtubules in the mammalian mitotic spindle. J Cell Biol. 147, 351–366 10.1083/jcb.147.2.351 10525540 PMC2174226

[67] Baumbach J., Novak Z.A., Raff J.W. and Wainman A. (2015) Dissecting the function and assembly of acentriolar microtubule organizing centers in *Drosophila* cells in vivo. PLoS Genet. 11, e1005261 10.1371/journal.pgen.1005261 26020779 PMC4447278

[68] Walczak C.E., Verma S. and Mitchison T.J. (1997) XCTK2: a kinesin-related protein that promotes mitotic spindle assembly in X*enopus laevis* egg extracts. J. Cell Biol. 136, 859–870 10.1083/jcb.136.4.859 9049251 PMC2132492

[69] Mahdipour M., Leitoguinho A.R., Zacarias Silva R.A., van Tol H.T., Stout T.A., Rodrigues G. et al. (2015) TACC3 is important for correct progression of meiosis in bovine oocytes. PLoS ONE 10, e0132591 10.1371/journal.pone.0132591 26168150 PMC4500572

[70] Brunet S., Dumont J., Lee K.W., Kinoshita K., Hikal P., Gruss O.J. et al. (2008) Meiotic regulation of TPX2 protein levels governs cell cycle progression in mouse oocytes. PLoS ONE 3, e3338 10.1371/journal.pone.0003338 18833336 PMC2556383

[71] Burgess S.G., Mukherjee M., Sabir S., Joseph N., Gutiérrez-Caballero C., Richards M.W. et al. (2018) Mitotic spindle association of TACC3 requires Aurora-A-dependent stabilization of a cryptic α-helix. EMBO J. 37 10.15252/embj.201797902 PMC5897774 29510984

[72] Cheeseman L.P., Booth D.G., Hood F.E., Prior I.A. and Royle S.J. (2011) Aurora A kinase activity is required for localization of TACC3/ch-TOG/clathrin inter-microtubule bridges. Commun. Integr. Biol. 4, 409–412 10.4161/cib.15250 21966557 PMC3181507

[73] Borisa A.C. and Bhatt H.G. (2017) A comprehensive review on Aurora kinase: small molecule inhibitors and clinical trial studies. Eur. J. Med. Chem. 140, 1–19 10.1016/j.ejmech.2017.08.045 28918096

[74] Bromberg J.F., Wrzeszczynska M.H., Devgan G., Zhao Y., Pestell R.G., Albanese C. et al. (1999) Stat3 as an oncogene. Cell 98, 295–303 10.1016/S0092-8674(00)81959-5 10458605

[75] Kanellos G. and Frame M.C. (2016) Cellular functions of the ADF/cofilin family at a glance. J. Cell Sci. 129, 3211–3218 10.1242/jcs.187849 27505888

[76] Li J., D'Angiolella V., Seeley E.S., Kim S., Kobayashi T., Fu W. et al. (2013) USP33 regulates centrosome biogenesis via deubiquitination of the centriolar protein CP110. Nature 495, 255–259 10.1038/nature11941 23486064 PMC3815529

[77] Hu S., Lu Y., Orr B., Godek K., Mustachio L.M., Kawakami M. et al. (2015) Specific CP110 phosphorylation sites mediate anaphase catastrophe after CDK2 inhibition: evidence for cooperation with USP33 knockdown. Mol. Cancer Ther. 14, 2576–2585 10.1158/1535-7163.MCT-15-0443 26304236 PMC4636444

[78] Stegmeier F., Rape M., Draviam V.M., Nalepa G., Sowa M.E., Ang X.L. et al. (2007) Anaphase initiation is regulated by antagonistic ubiquitination and deubiquitination activities. Nature 446, 876–881 10.1038/nature05694 17443180

[79] van Leuken R.J., Luna-Vargas M.P., Sixma T.K., Wolthuis R.M. and Medema R.H. (2008) Usp39 is essential for mitotic spindle checkpoint integrity and controls mRNA-levels of aurora B. Cell Cycle 7, 2710–2719 10.4161/cc.7.17.6553 18728397

[80] Vong Q.P., Cao K., Li H.Y., Iglesias P.A. and Zheng Y. (2005) Chromosome alignment and segregation regulated by ubiquitination of survivin. Science 310, 1499–1504 10.1126/science.1120160 16322459

[81] Song E.J., Werner S.L., Neubauer J., Stegmeier F., Aspden J., Rio D. et al. (2010) The Prp19 complex and the Usp4Sart3 deubiquitinating enzyme control reversible ubiquitination at the spliceosome. Genes Dev. 24, 1434–1447 10.1101/gad.1925010 20595234 PMC2895201

[82] Bretscher A., Edwards K. and Fehon R.G. (2002) ERM proteins and merlin: integrators at the cell cortex. Nat. Rev. Mol. Cell Biol. 3, 586–599 10.1038/nrm882 12154370

[83] Clucas J. and Valderrama F. (2014) ERM proteins in cancer progression. J. Cell Sci. 127(Pt 2), 267–275 10.1242/jcs.133108 24421310

[84] Carreno S., Kouranti I., Glusman E.S., Fuller M.T., Echard A. and Payre F. (2008) Moesin and its activating kinase Slik are required for cortical stability and microtubule organization in mitotic cells. J. Cell Biol. 180, 739–746 10.1083/jcb.200709161 18283112 PMC2265583

[85] Kunda P., Pelling A.E., Liu T. and Baum B. (2008) Moesin controls cortical rigidity, cell rounding, and spindle morphogenesis during mitosis. Curr. Biol. 18, 91–101 10.1016/j.cub.2007.12.051 18207738

[86] Fujiwara T., Bandi M., Nitta M., Ivanova E.V., Bronson R.T. and Pellman D. (2005) Cytokinesis failure generating tetraploids promotes tumorigenesis in p53-null cells. Nature 437, 1043–1047 10.1038/nature04217 16222300

[87] Ganem N.J., Storchova Z. and Pellman D. (2007) Tetraploidy, aneuploidy and cancer. Curr. Opin. Genet. Dev. 17, 157–162 10.1016/j.gde.2007.02.011 17324569

[88] Fukasawa K., Choi T., Kuriyama R., Rulong S. and Vande Woude G.F. (1996) Abnormal centrosome amplification in the absence of p53. Science 271, 1744–1747 10.1126/science.271.5256.1744 8596939

[89] Lopes C.A.M., Mesquita M., Cunha A.I., Cardoso J., Carapeta S., Laranjeira C. et al. (2018) Centrosome amplification arises before neoplasia and increases upon p53 loss in tumorigenesis. J. Cell Biol. 217, 2353–2363 10.1083/jcb.201711191 29739803 PMC6028540

[90] Cai Y., Liu Y.F., Yang H. and Lu H. (2009) [The p53-p21(waf1) pathway and centrosome amplification in oral squamous cell carcinomas]. Zhonghua Kou Qiang Yi Xue Za Zhi 44, 332–335 PMID:19953948

[91] Kawamura K., Izumi H., Ma Z., Ikeda R., Moriyama M., Tanaka T. et al. (2004) Induction of centrosome amplification and chromosome instability in human bladder cancer cells by p53 mutation and cyclin E overexpression. Cancer Res. 64, 4800–4809 10.1158/0008-5472.CAN-03-3908 15256449

[92] Meraldi P., Honda R. and Nigg E.A. (2002) Aurora-A overexpression reveals tetraploidization as a major route to centrosome amplification in p53−/− cells. EMBO J. 21, 483–492 10.1093/emboj/21.4.483 11847097 PMC125866

[93] Adon A.M., Zeng X.B., Harrison M.K., Sannem S., Kiyokawa H., Kaldis P. et al. (2010) Cdk2 and Cdk4 regulate the centrosome cycle and are critical mediators of centrosome amplification in p53-null cells. Mol. Cell. Biol. 30, 694–710 10.1128/MCB.00253-09 19933848 PMC2812235

[94] Marthiens V., Rujano M.A., Pennetier C., Tessier S., Paul-Gilloteaux P. and Basto R. (2013) Centrosome amplification causes microcephaly. Nat. Cell Biol. 15, 731–740 10.1038/ncb2746 23666084

[95] Tisato V., Voltan R., Gonelli A., Secchiero P. and Zauli G. (2017) MDM2/X inhibitors under clinical evaluation: perspectives for the management of hematological malignancies and pediatric cancer. J. Hematol. Oncol. 10, 133 10.1186/s13045-017-0500-5 28673313 PMC5496368

[96] Serçin O., Larsimont J.-C., Karambelas A.E., Marthiens V., Moers V., Boeckx B. et al. (2016) Transient PLK4 overexpression accelerates tumorigenesis in p53-deficient epidermis. Nat. Cell Biol. 18, 100–110 10.1038/ncb3270 26595384

[97] Shinmura K., Kurabe N., Goto M., Yamada H., Natsume H., Konno H. et al. (2014) PLK4 overexpression and its effect on centrosome regulation and chromosome stability in human gastric cancer. Mol. Biol. Rep. 41, 6635–6644 10.1007/s11033-014-3546-2 24981932

[98] Bailey A.W., Suri A., Chou P.M., Pundy T., Gadd S., Raimondi S.L. et al. (2018) Polo-like kinase 4 (PLK4) is overexpressed in central nervous system neuroblastoma (CNS-NB). Bioengineering (Basel) 5, E96 10.3390/bioengineering5040096 30400339 PMC6315664

[99] Coelho P.A., Bury L., Shahbazi M.N., Liakath-Ali K., Tate P.H., Wormald S. et al. (2015) Over-expression of Plk4 induces centrosome amplification, loss of primary cilia and associated tissue hyperplasia in the mouse. Open Biol. 5, 150209 10.1098/rsob.150209 26701933 PMC4703062

[100] He Y., Wang H., Yan M., Yang X., Shen R., Ni X. et al. (2018) High LIN28A and PLK4 coexpression is associated with poor prognosis in epithelial ovarian cancer. Mol. Med. Rep. 18, 5327–5336 10.3892/mmr.2018.9562 30365085 PMC6236221

[101] Li Z., Dai K., Wang C., Song Y., Gu F., Liu F. et al. (2016) Expression of polo-like kinase 4(PLK4) in breast cancer and its response to taxane-based neoadjuvant chemotherapy. J. Cancer 7, 1125–1132 10.7150/jca.14307 27326256 PMC4911880

[102] Wang J., Zuo J., Wang M., Ma X., Gao K., Bai X. et al. (2019) Pololike kinase 4 promotes tumorigenesis and induces resistance to radiotherapy in glioblastoma. Oncol. Rep. 41, 2159–2167 10.3892/or.2019.7012 30816483 PMC6412581

[103] Guizzunti G. and Seemann J. (2016) Mitotic Golgi disassembly is required for bipolar spindle formation and mitotic progression. Proc. Natl Acad. Sci. U.S.A. 113, E6590–E65E9 10.1073/pnas.1610844113 27791030 PMC5087019

[104] Gavilan M.P., Gandolfo P., Balestra F.R., Arias F., Bornens M. and Rios R.M. (2018) The dual role of the centrosome in organizing the microtubule network in interphase. EMBO Rep. 19, e45942 10.15252/embr.201845942 30224411 PMC6216252

[105] Suri A., Bailey A.W., Tavares M.T., Gunosewoyo H., Dyer C.P., Grupenmacher A.T. et al. (2019) Evaluation of protein kinase inhibitors with PLK4 cross-over potential in a pre-clinical model of cancer. Int. J. Mol. Sci. 20, 2112 10.3390/ijms20092112 PMC6540285 31035676

[106] Maiato H. and Logarinho E. (2014) Mitotic spindle multipolarity without centrosome amplification. Nat. Cell Biol. 16, 386–394 10.1038/ncb2958 24914434

[107] Jones L.A., Villemant C., Starborg T., Salter A., Goddard G., Ruane P. et al. (2014) Dynein light intermediate chains maintain spindle bipolarity by functioning in centriole cohesion. J. Cell Biol. 207, 499–516 10.1083/jcb.201408025 25422374 PMC4242835

